# Tocilizumab preserves renal function in rheumatoid arthritis with AA amyloidosis and end-stage kidney disease: Two case reports 

**DOI:** 10.5414/CN109971

**Published:** 2020-11-06

**Authors:** Makoto Fukuda, Naoki Sawa, Junichi Hoshino, Kenichi Ohashi, Miyazono Motoaki, Yoshifumi Ubara

**Affiliations:** 1 Nephrology Center, Toranomon Hospital Kajigaya, Kanagawa,; 2 Department of Pathology,; 3 Okinaka Memorial Institute for Medical Research, Toranomon Hospital, Tokyo,; 4 Department of Nephrology, Saga University Internal Medicine, Saga,; 5 Department of Pathology, Toranomon Hospital, Tokyo, and; 6 Department of Pathology, Yokohama City University, Graduate School of Medicine, Yokohama, Japan

**Keywords:** AA amyloidosis, tocilizumab, rheumatoid arthritis

## Abstract

Case 1: A 59-year-old Japanese woman with rheumatoid arthritis (RA) for 36 years was admitted for evaluation of deteriorating renal function. Her serum creatine was 4.2 mg/dL, and proteinuria was 6.5 g daily. Renal and duodenal biopsy revealed AA amyloidosis. After treatment with tocilizumab (a humanized anti-interleukin-6 receptor antibody), proteinuria decreased to 1.1 g daily. The patient’s renal function subsequently remained stable for 8 years. Case 2: A 71-year-old Japanese man with RA for 30 years was admitted due to deterioration of renal function. Serum creatine was 2.9 mg/dL, and urinary protein excretion was 0.06 g daily. Renal and duodenal biopsy identified AA amyloidosis. Tocilizumab was initiated, and his renal function remained stable for 6 years. The 2^nd^ duodenal biopsy showed a marked decrease of AA amyloid deposits. Conclusion: These two cases suggest that tocilizumab may preserve renal function in the setting of end-stage kidney disease and shift the point of no return for RA patients with AA amyloidosis and renal dysfunction.

## Introduction 

Secondary AA amyloidosis is a serious complication that occurs in patients with a long history of rheumatoid arthritis (RA), and is characterized by extracellular deposition of fibrils composed of serum amyloid A protein (SAA). Target organs for amyloid deposition in AA amyloidosis include the kidney, gastrointestinal tract, and heart. Renal biopsy is generally performed for diagnosis of AA amyloidosis [1]. Since tocilizumab, a humanized anti-interleukin (IL)-6 receptor antibody, and tumor necrosis factor (TNF) inhibitors have become available as standard treatment for RA, AA amyloidosis is now a treatable and preventable disease [2]. Tocilizumab has been reported to promote disappearance of gastrointestinal amyloid and marked improvement of amyloid heart disease as well as achieving remission of RA [3], but the effect of tocilizumab on the kidneys in AA amyloidosis has not been reported. Here we report 2 patients in whom progression of renal dysfunction was prevented after initiation of tocilizumab therapy. 

## Case reports 

### Case 1

In 2008, a 59-year-old Japanese woman was admitted for evaluation of renal disease. RA had been diagnosed at another hospital in 1972 when she presented with bilateral arthropathy of the hands, knees, ankles, and feet. Treatment was started with a combination of a gold preparation and nonsteroidal anti-inflammatory drugs (NSAIDs), but was not been effective. Prednisolone (PSL; 15 mg daily) and bucillamine (BUC; 200 mg daily) were started in 1987, but her disease remained active. Methotrexate (MTX; 5 mg daily) was started in 1995 but was discontinued because of nausea. In 2002, urinary protein was found to be positive by a dipstick urine test, and BUC was stopped. Then treatment was continued with PSL (5 mg/day) and loxoprofen (50 mg/day). However, urinary protein excretion increased in 2007, and serum creatinine (Cre) was elevated to 1.96 mg/dL. 

On admission, the patient was 154.2 cm tall and weighed 44.0 kg, with a blood pressure of 128/60 mmHg and temperature of 36.4 °C. Physical examination did not reveal any abnormalities of the heart and lungs. The joints of her hands, knees, ankles, and feet showed bilateral swelling and deformity. In addition, the lower extremities were edematous. Her cervical spine was unstable, with flexion causing numbness in the upper limbs. 

Laboratory findings were as follows: serum Cre was 4.2 mg/dL, the estimated glomerular filtration rate (eGFR) was 9.3 mL/min/1.73m^3^, C-reactive protein (CRP) was 0.9 mg/dL, and SAA was 43.2. In addition, rheumatoid factor (RF) was positive at 59 U/mL (normal: < 10), and cyclic citrullinated peptide (CCP) antibodies were positive at 218.5 (normal < 4.5). 24-hour urinary protein excretion was 6.5 g, and the urine sediment contained 1 – 5 red cells per high-power field (HPF). The disease activity score (DAS)-CRP was 7.1. Radiographs showed deformation of the finger and foot joints as well as atlantoaxial joint subluxation. Renal biopsy was performed for evaluation of her kidney disease. 

**Renal biopsy**

**Figure 1.**
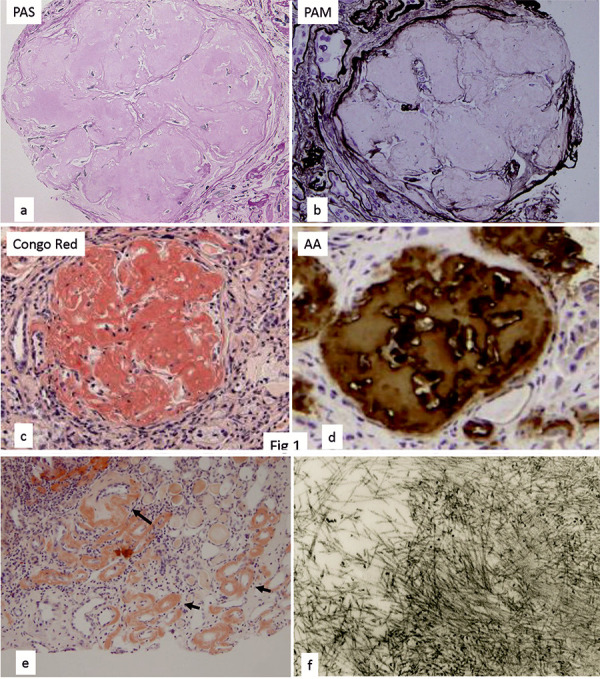
Light microscopic findings of the renal biopsy specimen of case 1. a: PAS stain; b: PAM stain; c: Congo red stain; d: AA amyloid; e: Congo red stain; f: electron microscopy.

**Figure 2.**
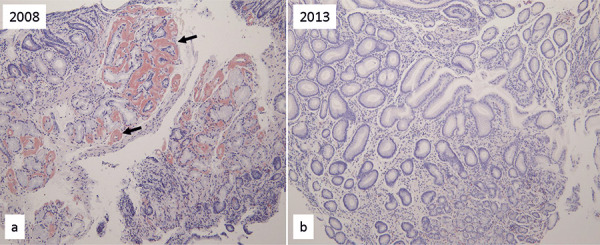
Gastroduodenal biopsy specimens of case 1. a: 2008; b: 2013. Arrow shows amyloid deposits.

Light microscopic examination of a biopsy specimen containing 4 glomeruli revealed global sclerosis in all 4. There was severe tubular atrophy, and tubulointerstitial fibrosis occupied ~ 95% of the entire renal cortex. All 4 glomeruli contained multinodular structures of amorphous material with a PAM-positive border. This material was positive for Congo-red and amyloid A, but was negative for κ and λ chains, β-2 microglobulin, and transthyretin (Figure 1). Electron microscopy showed randomly arranged fibrils measuring 8 – 12 nm in diameter corresponding to the amyloid deposits (Figure 1f). AA amyloidosis was diagnosed from these findings. In addition to the glomeruli, amyloid deposits were mainly observed in the interlobular artery walls and tubulointerstitium (Figure 1e). Endoscopic biopsy of the stomach, duodenum, and colon revealed AA-positive deposits in the small arteries and tissues of the submucosal layer (Figure 2a). 

**Clinical course**

**Figure 3.**
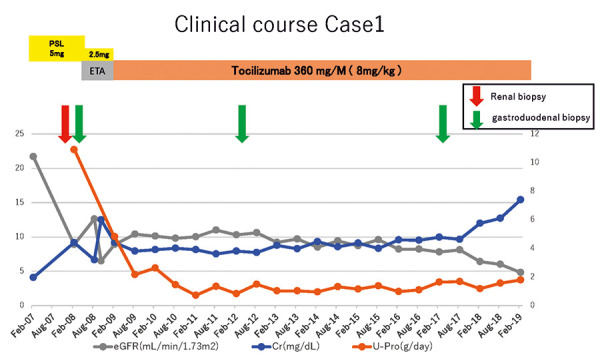
Clinical course of case 1. ETA = etanercept; PSL = prednisolone.

PSL was discontinued, and administration of a soluble tumor necrosis factor (TNF) receptor inhibitor (etanercept; 25 mg every 2 weeks) was started in May 2008, but it was not effective. By September 2008, Cre was increased to 6.0 mg/dL. She underwent surgery to prepare an arteriovenous fistula for hemodialysis. Etanercept was discontinued, and a humanized anti-interleukin-6 receptor antibody (tocilizumab; 8 mg/kg = 360 mg/month) was started in February 2009. After 3 months, her CRP decreased to 0.0 mg/dL, and the DAS28-CRP sore was 2.12. After 2 years of tocilizumab therapy, urinary protein excretion was decreased to 1.1 g/day, and Cre was 4.0 mg/dL. Subsequently, Cre remained in the range of 4.5 – 5.0 mg/dL until December 2017. While Cre increased to 7.1 mg/dL after initiation of treatment with denosumab (a human monoclonal antibody that binds to receptor activator of NFκB ligand) for osteoporosis in October 2018, it remained at 7.0 mg/dL in June 2019 (Figure 3). 

**Figure 4. Figure4:**
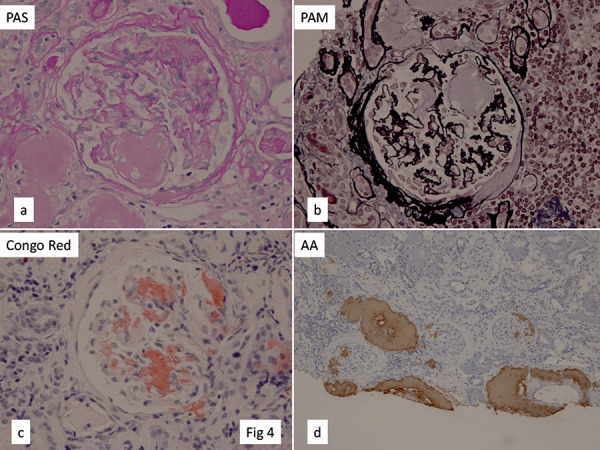
Light microscopic findings of the first renal biopsy specimen of case 2. a: PAS stain; b: PAM stain; c: Congo red stain; d: AA amyloid.

Gastroduodenal biopsy was performed in May 2013 and May 2017. On both occasions, no amyloid deposits were detected in the submucosal blood vessels [Fig Figure4](Figure 2b). 

### Case 2

In 2012, a 71-year-old man was admitted to our hospital for evaluation of renal dysfunction. RA had been diagnosed in 1982 when he developed arthropathy of the bilateral hands and feet. Treatment was started with BUC and salazosulfapyridine (SSAP). In December 2009, MTX was added at 4 mg weekly, but it was discontinued because of anemia in January 2010 and PSL (5 mg/day) was added from February 2010. Etanercept was started in December 2010, and treatment with abatacept (the Fc region of immunoglobulin IgG1 fused to the extracellular domain of cytotoxic T lymphocyte-associated antigen 4 (CTLA-4)) was commenced in March 2011. Subsequently, his renal function began to decline. 

On admission, he was 167.1 cm tall and weighed 53.0 kg, with a blood pressure of 115/83 mmHg and a temperature of 36.9 °C. The joints of both hands were swollen and deformed, and there was edema of the lower extremities. 

Laboratory findings were as follows: Cre was 2.8 mg/dL, eGFR was 19.1 mL/min/1.73 m^2^, CRP was 0.1 mg/dL, anti-CCP antibody was 590.0 U/mL, and SAA was 16.5 μg/mL. Urinary protein excretion was 0.06 g/day, and the urine sediment contained < 1 RBC per HPF. The DAS-CRP score was 2.6. 

**First renal biopsy**

**Figure 5.**
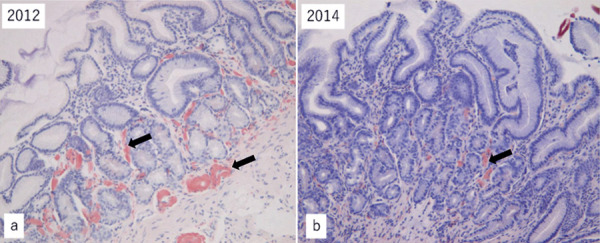
Gastroduodenal biopsy specimens of case 2. a: 2012; b: 2014. Arrow shows amyloid deposits.

Light microscopic examination of a renal biopsy specimen containing 25 glomeruli revealed global sclerosis in 7. Tubular atrophy was severe, and tubulointerstitial fibrosis occupied ~ 80% of the entire renal cortex. Amorphous deposits were detected in the glomeruli, glomerular vascular pole, interlobular arteries, and tubulointerstitial region. These deposits were positive for Congo red and amyloid A, but were negative for κ and λ chains, β-2 microglobulin, and transthyretin (Figure 4). Biopsy of the stomach and duodenum also revealed AA amyloid deposits in the small arteries and tissues of the submucosal layer (Figure 5a). These findings led to diagnosis of AA amyloidosis. 

**Clinical course**

**Figure 6.**
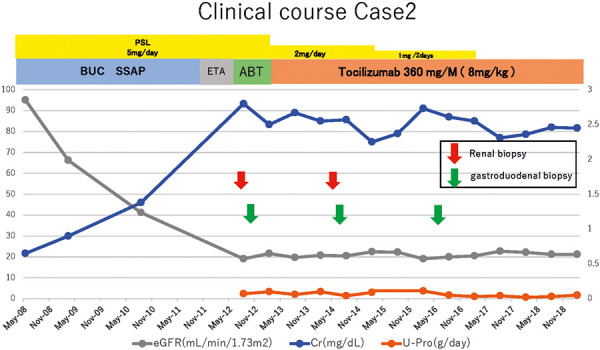
Clinical course of case 2. TCZ = tocilizumab; SSAP = salazosulfapyridine, ABT = abatacept; ETA = etanercept; PSL = prednisolone.

PSL was tapered to 2 mg/day, and abatacept was discontinued, while tocilizumab was started in August 2012. The DAS28-CRP core decreased to 1.5. PSL was discontinued in February 2017. Cre decreased from 2.82 to 2.5 mg/dL and remained at 2.3 mg/dL in June 2019 (Figure 6). 

**Second renal biopsy**

The second renal biopsy was performed in April 2014. Light microscopic examination of a renal specimen containing 36 glomeruli revealed global sclerosis in 20. Tubulointerstitial fibrosis occupied ~ 80% of the entire cortex. Amyloid deposits in the glomeruli and small arteries were unchanged or slightly increased compared with the first biopsy. However, repeat gastroduodenal biopsy showed a marked decrease of amyloid deposition (Figure 5b).****

## Discussion 

Okuda et al. [4] reviewed 199 patients in whom AA amyloidosis was diagnosed from January 2012 to December 2014. The primary disease was rheumatoid arthritis in 60.3%, unclassified autoimmune disease in 11.1%, malignancy in 7.0%, inflammatory bowel disease in 4.5%, chronic infection in 4.5%, and Castleman’s disease in 4.0%. Major clinical manifestations were moderate renal dysfunction in 46.2%, moderate proteinuria in 30.7%, and diarrhea in 32.2%. Diagnosis was made by gastroduodenal mucosal biopsy in 66.3%, renal biopsy in 22.1%, myocardial biopsy in 5.5%, and abdominal fat biopsy in 4.0%. Suppressing the production of SAA is important for control of AA amyloidosis. SAA is produced in the liver due to stimulation by pro-inflammatory cytokines such as IL-6, TNF, and IL-1, so it is desirable to reduce the levels of such cytokines to decrease SAA. Although SAA levels are reduced by TNF-α inhibitors and agents targeting CTLA-4, such as abatacept, normalization is not always achieved. On the other hand, it has been reported that treatment with IL-6 inhibitors can normalize the SAA level in most patients [2, 5]. Among the renal lesions complicating RA, reports of membranoproliferative glomerulonephritis, membranous nephropathy, and AA amyloidosis are frequent [6]. Regarding the association between IL-6 and histologic kidney damage, experiments in mice have shown that IL-6 promotes glomerular hypertrophy and mesangial proliferation [7]. Kuroda et al. [8] administered TNF-α inhibitors to 14 patients with AA amyloidosis secondary to RA (infliximab in 10 patients and etanercept in 4 patients) and reported a decrease of both CRP and SAA. Nine of the 14 patients underwent gastrointestinal mucosal biopsy twice and showed a decrease of amyloid deposition. With regard to renal function, improvement of creatinine clearance (CCr) was noted in 4 of the 14 patients, while it was unchanged in 5 patients and showed deterioration in 3 patients. Urinary protein excretion was decreased in 3 of the 14 patients, while it was unchanged in 6 and increased in 3. In addition, Ishii et al. [9] reported significant regression of gastric and duodenal mucosal amyloid deposits within 4 months of starting treatment with prednisolone and etanercept in RA patients who had AA amyloidosis, and the anti-inflammatory effect of the TNF-α inhibitor was reported to promote regression of amyloid deposits. In addition, Yamada et al. [10] reported an RA patient with nephrotic syndrome and AA amyloidosis on renal biopsy, in whom proteinuria improved dramatically soon after initiation of tocilizumab. 

Thus, there have been reports about short-term improvement of renal function and urinary protein excretion (Table 1), but there have been no previous reports on maintenance of renal function for a long period (11 years and 7 years in our 2 cases). In 27 patients with refractory RA who had already been treated with at least one biological preparation, Addimanda et al. [11] examined various parameters at 3 months and 6 months after starting tocilizumab therapy. Compared with baseline, they found reduction of CRP, RF, anti-CCP antibody, ESR, and DAS28, and the steroid dose could also be decreased. Tocilizumab has also been reported to be effective for patients who were resistant to other biological agents. Moreover, Courties et al. [12] evaluated urinary protein excretion, GFR, and the tocilizumab continuation rate in 12 patients with histologically confirmed AA amyloidosis. Eight patients had RA, 6 had a history of TNF-α inhibitor therapy, and 2 were already on dialysis. Urinary protein excretion was 5 ± 3.3 g/day, and GFR was 53.6 at initiation of tocilizumab. After a mean follow-up period of 13 months, tocilizumab administration could be continued in 7 out of 12 patients (58%). Tocilizumab was effective for controlling disease activity in 6 of the 8 patients with RA. Renal amyloid showed progression in 3 patients and was stable in 3, while urinary protein excretion decreased steadily. Among the 6 patients with RA who had previously been treated with TNF-α inhibitors, amyloidosis improved in 1 and was stable in 3. 

In patients with IgA nephropathy, the “point of no return (PNR)”, after which progression to end-stage renal disease becomes inevitable, has been reported to be a Cre of 2.0 mg/dL (equivalent to an eGFR of 30 – 35 mL/min/1.73m^2^) [13]. However, our 2 cases suggest that tocilizumab treatment might be able to preserve residual renal function in RA patients with AA amyloidosis and end-stage kidney disease, thus changing the PNR for RA with AA amyloidosis because renal function of both patients (eGFR: 19.1 and 9.3 mL/min/1.73m^2^) was maintained for 6 years and 8 years after starting tocilizumab, respectively. 

## Funding 

This study was funded by the Okinaka Memorial Institute for Medical Research. 

## Conflict of interest 

The authors declare no competing financial interest. 

The authors also declare that they have no conflict of interest. The patients gave written informed consent to the publication of the details of their cases. 

**Table 1.** Report of rheumatoid arthritis successfully treated with biologics.

References	Number of cases	Biologics	First biopsy organ	First biopsy organ lesions	Repeat biopsy organ	Repeat biopsy organ lesions	Number of kidney function improvement or stable cases
[8]	4	Infliximab	Gastrointestinal mucosa	Congo-red-positive deposits were seen in gastric mucosal and submucosal lesions	Gastrointestinal mucosa	The percentage of areas of amyloid deposition was decreased	4
[8]	10	Etanercept	Gastrointestinal mucosa	Congo-red-positive deposits were seen in gastric mucosal and submucosal lesions	Gastrointestinal mucosa	The percentage of areas of amyloid deposition was decreased	7
[9]	1	Etanercept	Gastrointestinal mucosa	Heavy deposition of amyloid was visible in lamina propria and vascular walls of all specimens	Gastrointestinal mucosa	Amyloid deposition was decreased	1
[10]	1	Tocilizumab	Kidney	Deposition of amyloid was seen in the mesangial area and vascular wall	n/a	n/a	1
[12]	3	Tocilizumab	Salivary gland	Deposition of amyloid was seen	n/a	n/a	2
[12]	5	Tocilizumab	Kidney	Deposition of amyloid was seen	n/a	n/a	5

n/a = not available**.**
